# Magnetic resonance imaging and dynamic X-ray’s correlations with dynamic electrophysiological findings in cervical spondylotic myelopathy: a retrospective cohort study

**DOI:** 10.1186/s12883-020-01945-4

**Published:** 2020-10-06

**Authors:** Zhengran Yu, Kaiyuan Lin, Jiacheng Chen, Kuan-Hung Chen, Wei Guo, Yuhu Dai, Yuguang Chen, Xuenong Zou, Xinsheng Peng

**Affiliations:** Guangdong Provincial Key Laboratory of Orthopaedics and Traumatology, Orthopaedic Research Institute/Department of Spine Surgery, The First Affiliated Hospital of Sun Yat-sen University, Guangzhou, 510080 P. R. China

**Keywords:** Cervical spondylotic myelopathy, Dynamic-somatosensory-evoked potentials, Magnetic resonance imaging, Dynamic X-ray, Cervical segmental instability

## Abstract

**Background:**

Dynamic somatosensory evoked potentials (DSSEP) can be used to disclose abnormalities of ascending sensory pathways at dynamic positions and diagnose cervical spondylotic myelopathy (CSM). However, radiographic tests including magnetic resonance imaging (MRI) and dynamic X-ray are used much more widely in the management of CSM. Our study aims to clarify the correlations between several radiographic parameters and the DSSEP results, and further determine their reliability with clinical data.

**Methods:**

We retrospectively enrolled 38 CSM patients with surgical intervention. DSSEP tests were performed before surgery. Amplitude ratios of DSSEP N13 and N20 waves at extension and flexion were calculated and recorded as N13_E, N20_E, N13_F, N20_F, respectively. Baseline severity was evaluated with the modified Japanese Orthopedic Association (mJOA) score and the Nurick grades. Prognosis was evaluated based on the 2-year recovery rate. Sagittal diameter and transverse areas of the cord and canal were measured and the the compressive ratios at the compressed site (Compression_Ratio), central (Central_Ratio), and 1/4-lateral points (1/4-Lateral_Compression_Ratio), and spinal cord/Canal Area Ratio were calculated. The intramedullary T2 hyperintensity patterns (Ax-CCM types) were also collected from MRI axial images. Dynamic X-rays were used to test for segmental instability of the cervical spine. The correlations between radiologic findings, DSSEP data, and clinical assessments were investigated.

**Results:**

We found that DSSEP N13_E and N13_F correlated with the Compression_Ratio, Central_Ratio, 1/4-Lateral_Compression_Ratio (Pearson, *p* < 0.05) and Ax-CCM types (ANOVA, p < 0.05) in MRI axial images and cervical segmental instability in dynamic X-ray (t-test, *p* < 0.05). Apart from the 1/4-Lateral_Compression_Ratio, these radiographic parameters above also correlated with the baseline clinical assessments (Spearman or ANOVA or t-test, *p* < 0.05) and postoperative recovery rate (Pearson or ANOVA or t-test, p < 0.05).

**Conclusions:**

We found that the preoperative Compression_Ratio, Central_Ratio and 1/4-Lateral_Compression_Ratio in MRI and cervical segmental instability in dynamic X-ray could reflect the dynamic neural dysfunction of the spinal cord. Different Ax-CCM types corresponded to different DSSEP results at extension and flexion, suggesting divergent pathophysiology. These radiographic parameters could help evaluate disease severity and predict postoperative prognosis.

## Background

In cervical spondylotic myelopathy (CSM) patients, cervical myelopathy is caused by both static compressions as well as dynamic compression during cervical motion (flexion/extension) [1]. At a neutral position, static compressions can be caused by herniated discs, spondylotic spurs, ossification of the posterior longitudinal ligament and hypertrophy of the ligamentum flavum [2]. During flexion, the spinal cord may become compressed against osteophytic spurs and protruding discs [3–5]. With hyperextension, the ligamentum flavum or laminae can pinch the spinal cord posteriorly, causing a pincer effect [6]. Therefore, the mechanical consequences of dynamic compression on the spinal cord should not be neglected.

Magnetic resonance imaging (MRI) is widely used in the management of CSM. There have been some studies on the associations between MRI factors, including relating cord compression and signal changes of the spinal cord on T1- and T2-weighted imaging and clinical symptoms and recovery after surgery [7]. From the many methods mentioned, the absolute value of sagittal diameter and transverse area of the spinal cord, the area ratio between the spinal cord and spinal canal, Compression_Ratio (sagittal diameter at most compressed site / transverse diameter of the spinal cord observed on axial T2WI), Central_Compression_Ratio (central sagittal diameter / transverse diameter) and 1/4-Lateral_Compression_Ratio (1/4-lateral anteroposterior diameter / transverse diameter) have been used regularly and are reported in the literature [7]. The size and pattern of intramedullary spinal cord signal intensity changes are also related to the severity of impairment in patients with CSM [8]. Dynamic X-ray is frequently used to evaluate cervical segmental instability among CSM patients and its results also correlate with the preoperative symptoms and surgical outcomes [9, 10]. However, correlating imaging findings with the clinical picture is complicated by the increasingly wide-range of measurements on radiographs including MRI and dynamic X-ray. In addition, there remain fundamental knowledge gaps surrounding the relationship of neural functional change at dynamic positions with findings seen on static radiographs. Due to the difficulty of quantifying the extent of spinal cord dysfunction from physiological findings, electrophysiological findings were used.

Somatosensory evoked potentials (SSEP) have been utilized as useful neurophysiological indicators to detect objective functional abnormalities of the spinal cord [11, 12]. It has been suggested that the spinal N13 response reflected segmental cord dysfunction [13], and have higher sensitivity in diagnosing CSM than the scalp N20 waves [14]. On the basis of these studies, we developed the dynamic somatosensory evoked potentials (DSSEP), which is performed at neutral, extension, and flexion positions [15]. The percent change of DSSEP N13 amplitudes has been proven as an effective parameter for diagnosing CSM with high sensitivity and specificity [15]. In this study, we investigated the effectiveness of different MRI parameters and dynamic X-ray for assessing the dynamic neural dysfunction measured with DSSEP. We also correlated these radiographic results with baseline clinical conditions and postoperative recovery to further testify their reliability.

## Methods

### Patient cohort

The Human Ethics Committee of the First Affiliated Hospital of Sun Yat-sen University approved the trial, and informed consent was acquired before the DSSEP tests. The single-center retrospective study included 38 CSM patients with preoperative DSSEP, MRI, and dynamic X-ray tests and later had surgery at the Spine Surgery Department, First Affiliated Hospital of Sun Yat-sen University (Guangzhou, China) between 2015 to 2017. All participants had at least two years follow-up. Patients with congenital spinal deformity, a history of stroke, surgical treatment, peripheral neurological disease, ulnar or carpal tunnel syndrome, or diabetes were excluded. Demographic data collected included sex, age, and critical comorbidities. Measures of neurological disability included the modified Japanese Orthopaedic Association (mJOA) score [16] and Nurick grade [17]. Ataxia were defined as the absence of dysarthria, nystagmus, abnormal eye movements, and the presence of abnormal joint position senses and a positive Romberg sign with gait disorder in this study [18]. The 2-year postoperative mJOA scores were used to calculate the recovery rates with the Hirabayashi method [19]: Recovery rate = [postoperative mJOA score - preoperative mJOA score] / [21 - preoperative mJOA score] × 100%.

### Realization and measurement of DSSEP

**Fig. 1:**
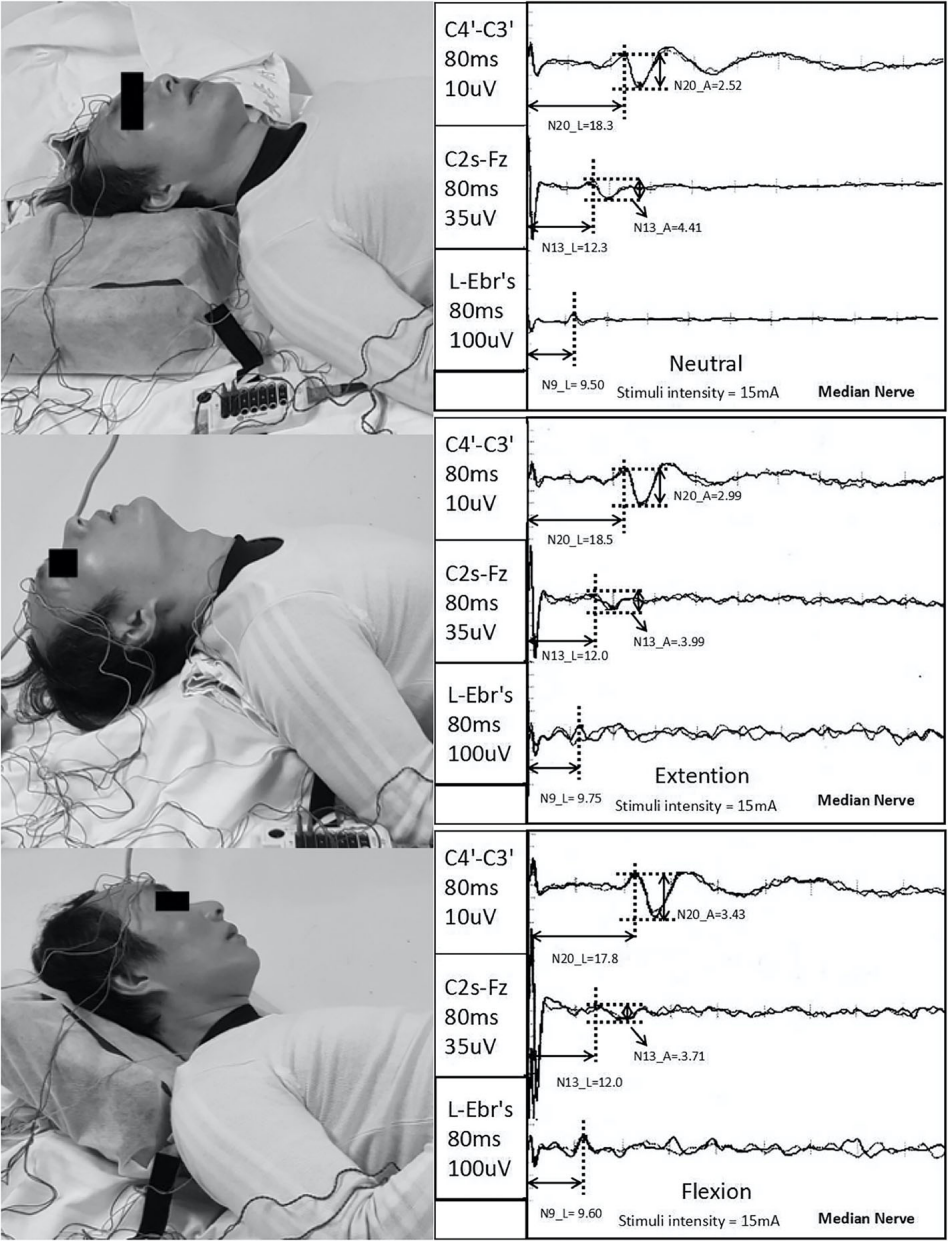
DSSEP test illustration: Left: Cervical spine in neutral, 20° extension and 35° flexion positions. Right: DSSEP results of the left median nerve of this CSM patient. The latencies of N9, N13 and N20, amplitudes of N13 and N20 are shown in the figure

An electrophysiological monitoring system (Nicolet Endeavor CR) was used to elicit and record the SSEPs. Median and ulnar nerve SEPs were examined using established methods [20]. The ulnar and median nerves were stimulated with 0.2 msec square wave pulses delivered at a rate of 5 Hz with an impedance of less than 5000 W. Visible contraction of the abductor pollicis brevis muscle was used as an indicator of appropriate stimulus intensity. SSEPs were recorded using subdermal needle electrodes placed at Erb’s point ipsilateral to the stimulation (Erb’s) and spinous processes of the second cervical vertebrae (C2s-Fz) with the reference electrode at Fz. Another two electrodes were placed at the sensory cortex of the left and right hemisphere (about 1 cm medial-posterior to the C3 and C4 points according to the international 10–20 system, marked as C4’-C3’). The SSEPs were measured with the cervical spine in a neutral position. Patients were then positioned at about 35° flexion and after which at about 20° extension of the cervical spine using a device for elevating the head and neck with minimal discomfort to the subject (Fig. 1). To confirm the reproducibility of the SSEPs, each measurement was carried out at least three times by a spine surgeon and two electrophysiologists.

The amplitudes for each recording position labeled Erb’s, C2s-Fz, and C4’-C3’ were recorded as N9, N13, and N20, respectively. We also simultaneously recorded the response latencies. Both the ulnar and median nerve stimuli were recorded. According to our previous study [15], only the N13 amplitudes and their percent changes have been used for statistical analysis in this study.

### Imaging methods and analysis protocol

All MR examinations were performed with a 3.0-T MR imager (Siemens Trio) with the patients lying in a supine position on a spine-array coil. The authors evaluated compressed spinal cords using standard imaging sequences. T1 and T2-weighted spin-echo sagittal sequences were performed using the following parameters: T1: TR 650, TE 10, Slice thickness 3 mm, dist 0.3 mm gap, FA 150°, TA 1′36″, Matrix 320 × 224; T2: TR 2800, TE 97, Slice thickness 3 mm, dist 0.3 mm gap, FA 160°, TA 2′10″, Matrix 384 × 308. T1 and T2-weighted spin-echo axial sequences were performed using the following parameters: T1: TR 466, TE 11, Slice thickness 4 mm, dist 0.4 mm gap, FA 120°, TA 2′26″, Matrix 256 × 205; T2: TR 3260, TE 90, Slice thickness 4 mm, dist 0.4 mm gap, FA 150°, TA 1′52″, Matrix 320 × 256.

**Fig. 2:**
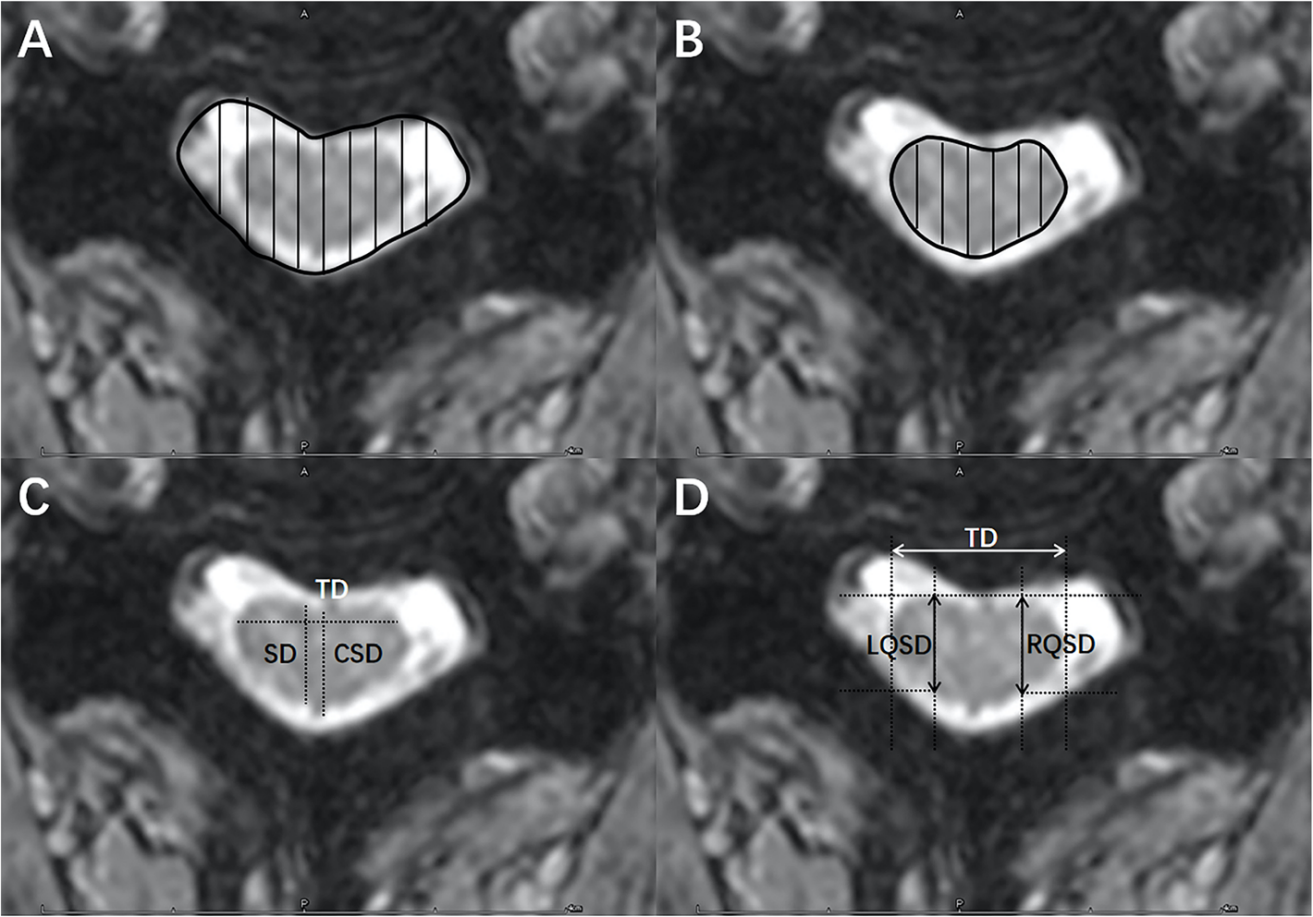
MRI measurements illustration. **a**: transverse spinal canal area (subarachnoid space); **b**: Transverse spinal cord area; **c**: Central_Ratio and Compression_Ratio are calculated as follows: Central_Ratio = CSD / TD, Compression_Ratio = SD / TD. (SD = sagittal spinal cord diameter, TD = transverse spinal cord diameter, CSD = Central sagittal diameter of the spinal cord); **d**: 1/4-Lateral_Compression_Ratio is calculated as follow: 1/4-Lateral_Compression_Ratio = (LQSD + RQSD) / 2TD. (RQSD = 1/4-lateral anteroposterior diameter on the right side, LQSD = 1/4-lateral anteroposterior diameter on the left side)

The measurements of the cervical spinal cord in MRI T2WI axial images were performed with Photoshop CC (Adobe, San Jose, California). The transverse area of the spinal canal and spinal cord were respectively measured. The Cord/Canal_Area_Ratio was defined as follows: Cord/Canal_Area_Ratio = Area of the spinal cord / Area of the canal. The linear parameters including transverse diameter (TD), central sagittal diameter (CSD) and sagittal diameter (SD) were measured, and the Central_Ratio, Compression_Ratio and 1/4-Lateral_Compression_Ratio were calculated (Fig. 2). The Ax-CCM classification system is defined as follows [8]: type 0 = normal signal intensity of spinal cord without any intramedullary T2 hyperintensity, type 1 = diffuse pattern of intramedullary T2 hyperintensity occupying more than two-thirds of the axial dimension of the spinal cord with an obscure and faint border, type 2 = focal intramedullary T2 hyperintensity with an obscure and faint border, type 3 = focal intramedullary T2 hyperintensity with a well-defined and distinct margin.

Extension-Flexion (dynamic) X-ray studies of all 38 patients were analyzed. Cervical segmental instability was determined according to the White-Panjabi standard [9]: (1) translational instability: more than 3.5 mm horizontal displacement of one vertebra in relation to an adjacent vertebra, either anteriorly or posteriorly; (2) rotational instability: more than 11-degree rotational difference to that of either adjacent vertebrae. Patients with cervical segmental instability in dynamic X-rays were classified as the “Unstable” group, and others were classified as the “Stable” group.

The MRI and dynamic X-ray films of the cervical spines were studied three times by two spine surgeons and a radiologist, and the mean values were used. The measurements were performed in a blind fashion, so that patients’ names, clinical characteristics, and the results of the electrophysiological studies were unknown to the observers.

### Statistical analysis

Amplitudes of left and right sides of the DSSEP N13 or N20 waves were averaged first. Then the N13 or N20 amplitude ratios at extension and flexion were calculated and recorded as N13_E, N20_E, N13_F, N20_F, respectively. We defined the DSSEP amplitude ratio as the following: amplitude ratio = (Amplitude_extension or Amplitude_flexion ÷ Amplitude_neutral) × 100%. For absent N13 or N20 waves, their latencies were excluded and their amplitudes were set as the baseline value (0 mV) for statistical analysis [14]. For CSM patients whose DSSEP waves at neutral position were lost, amplitude ratios of their corresponding DSSEP waves were also set as 0.

Student t-test, Pearson or Spearman correlation method and one-way analysis of variance analysis (ANOVA) were applied in this study. All data were presented as mean ± SD, and a *P*-value < 0.05 was considered significant. The statistical software R (R version 3.6.0) was used for statistical analysis.

## Results

### DSSEP N13_E and N13_F are sensitive parameters for evaluating the CSM

Demographic data was shown in Table 1. The preoperative mJOA score and Nurick grade were 13.95 ± 2.01 (range 9–17) and 2.61 ± 1.18 (range 1–4) respectively. There were 20 (52.6%) patients presented ataxia preoperatively. The two-year postoperative mJOA score and recovery rate were 16.89 ± 2.44 (range 12–20) and 45.6% ± 21.9% (range 0–80%) respectively (Table 1). We recorded latencies for N9, N13 and N20 and amplitudes for N13 and N20 during cervical flexion and extension. Four (10.5%) patients’ N13 or N20 waves were lost at all three positions, three (7.9%) were lost at both dynamic positions, and six (15.8%) were lost at either the extension or flexion position. We found that the DSSEP N13 amplitudes were significantly higher at the neutral position than that at both the extension (t-test, *p* < 0.001) and flexion (t-test, *p* < 0.01) positions (Table 2). We calculated the amplitude ratios, the N13_E and N13_F were 0.72 ± 0.34 and 0.73 ± 0.43 respectively, and the N20_E and N20_F were 0.97 ± 0.29 and 0.97 ± 0.29 respectively. The DSSEP N20 amplitudes, and N13 and N20 latencies did not change significantly during extension or flexion (Table 2).

**Table 1:** Summary of demographics and clinical data of 38 CSM patients

Variable	Measurement
No. males	21 (55.3%)
Mean age (yr)	53.6 (range 22–82)
Preoperative clinical assessment	
mJOA score	13.95 ± 2.01 (range 9–17)
Nurick grades	2.61 ± 1.18 (range 1–4)
No. Hoffman sign	24 (64.9%)
No. Leg hyperreflexia	13 (35.1%)
No. Ataxia	20 (52.6%)
2-Year Postoperative clinical assessment	
mJOA score	16.89 ± 2.44 (range 12–20)
Recovery rate	45.6% ± 21.9% (range 0–80%)
Compression levels (n) at MRI	
Stenotic levels	C3/4 (15)
	C4/5 (21)
	C5/6 (30)
	C6/7 (12)
	C7/T1 (1)
Most stenotic level	C3/4 (4)
	C4/5 (8)
	C5/6 (22)
	C6/7 (4)
No. (%) undergoing each procedure	
Anterior	27 (71.1%)
Posterior	7 (18.4%)
Combined anterior-posterior	4 (10.5%)

**Table 2:** Results of DSSEP findings in neutral and dynamic positions^†^

	Neutral		Extension		Flexion	
	N13	N20	N13	N20	N13	N20
Latency (ms)	13.2 ± 1.1	18.88 ± 1.09	13.25 ± 1.01	19.06 ± 1.01	13.14 ± 1.07	18.95 ± 1.07
p_value ^‡^			0.15	0.09	0.43	0.3
Amplitude (uV)	2.66 ± 1.42	2.7 ± 1.59	2.17 ± 1.29	2.53 ± 1.3	2.18 ± 1.59	2.56 ± 1.54
p_value ^‡^			0.000017 ***	0.29	0.0026 **	0.25
Amplitude Ratio ^§^			0.72 ± 0.34	0.97 ± 0.29	0.73 ± 0.43	0.97 ± 0.29
Absent waves(Left, Right) ^¶^	4 (4,4)	0	8 (7,6)	2 (1,2)	10 (7,9)	1 (1,1)

** *p*_value< 0.01; *** *p*_value< 0.001

^†^ For absent N13 or N20 waves, their latencies were excluded and amplitudes were set as the baseline value (0 mV) for statistical analysis

^‡^ The p_values were calculated with the student’s t-test by comparing the DSSEP value at dynamic (extension or flexion) position with neutral position

^§^ Each patients’ amplitude ratios were calculated with the following method: Amplitude Ratio = amplitude at dynamic (extension or flexion) position ÷ amplitude at neutral position

^¶^ The number of patients whose waves at either left or right side at specific position was lost. The number of absent waves at left and right sides are respectively listed in the bracket

We compared the DSSEP results with our clinical assessments. We found that the N13 DSSEP amplitude ratios at both extension and flexion positions positively correlated with the baseline clinical symptoms and postoperative outcomes: results having positive correlation with mJOA scores (Spearman, N13_E: R = 0.40, *p* = 0.014; N13_F: R = 0.35, *p* = 0.031), negative correlation with Nurick grades (Spearman, N13_E: R = -0.34, *p* = 0.039; N13_F: R = -0.39, *p* = 0.015) and positive correlation with recovery rates (Pearson, N13_E: R = 0.37, *p* = 0.021; N13_F: R = 0.54, *p* = 0.0004). However, only the N13_E in ataxic patients was significantly lower than that in patients without ataxia (0.61 ± 0.39, 0.85 ± 0.22, t-test, *p* = 0.023). The N13_F did not show significant difference between the ataxia and non-ataxia groups (0.61 ± 0.43, 0.82 ± 0.34, t-test, *p* = 0.11).

### The radiographic findings and their correlations with N13_E and N13_F

The preoperative transverse area of the spinal cord and spinal canal obtained with MRI were 69.95 ± 19.01 and 111.79 ± 38.64 mm^2^ respectively, and the Cord/Canal Area Ratio was 0.66 ± 0.16. The sagittal diameter of the spinal cord was 4.77 ± 1.09 mm. The Compression_Ratio, Central_Ratio, and 1/4-Lateral_Compression_Ratio were 0.35 ± 0.08, 0.44 ± 0.1, and 0.34 ± 0.08 respectively.

**Fig. 3:**
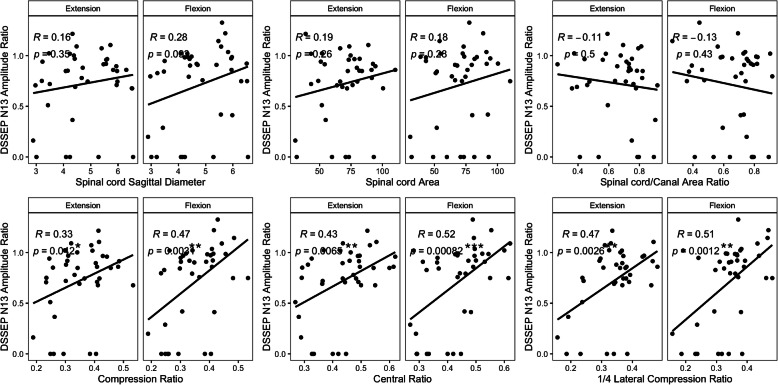
Pearson correlations between the DSSEP N13 amplitude ratios and MRI measurements. The DSSEP N13 amplitude ratios were statistically correlated with the Compression_Ratio, Central_Ratio and 1/4-Lateral_Compression_Ratio at both extension and flexion, while there were no correlations between the DSSEP results and spinal cord sagittal diameter, spinal cord area or spinal cord/canal area ratio. * *p*_value< 0.05; ** *p*_value< 0.01; *** *p*_value< 0.001

Both the N13_E and N13_F positively correlated with Compression_Ratio (Pearson, N13_E: R = 0.33, *p* = 0.042; N13_F: R = 0.47, *p* = 0.003), Central_Ratio (Pearson, N13_E: R = 0.43, *p* = 0.007; N13_F: R = 0.52, *p* = 0.0008) and 1/4-Lateral_Compression_Ratio (Pearson, N13_E: R = 0.47, p = 0.003; N13_F: R = 0.51, *p* = 0.001) measured in MRI axial images. There was no correlation between DSSEP N13 amplitude ratios and spinal cord area, Cord/Canal Area Ratio or Sagittal Diameter (Fig. 3).

**Fig. 4:**
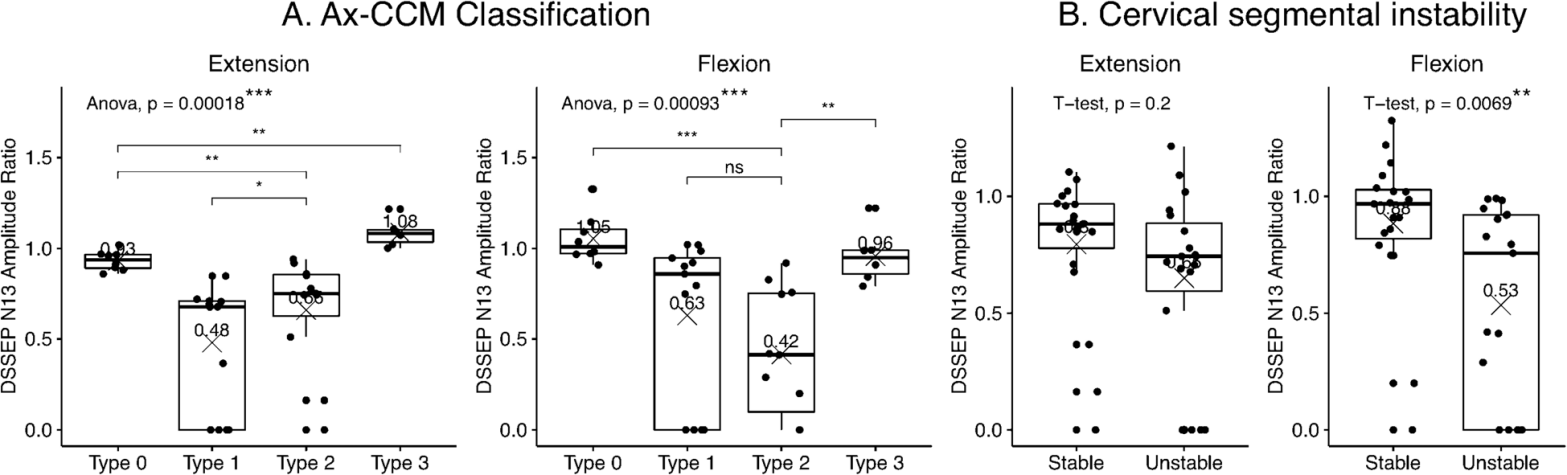
**a**. Boxplots showing the ANOVA results of the four groups based on the Ax-CCM classification system. Differences between groups were analyzed with post hoc t-test when the ANOVA showed significant difference among groups. **b**. Student’s t-test results of patients’ N13_E or N_13F in “Stable” and “Unstable” groups based on dynamic X-ray studies. * *p*_value< 0.05; ** *p*_value< 0.01; *** *p*_value< 0.001

The number of 8, 13, 11 and 6 patients were classified as the Ax-CCM Type 0, Type 1, Type 2 and Type 3 respectively. The mean N13_E for each Ax-CCM group was 0.93 ± 0.05, 0.48 ± 0.34, 0.66 ± 0.30, 1.08 ± 0.07 respectively, and mean N13_F was 1.05 ± 0.12, 0.63 ± 0.43, 0.42 ± 0.33, 0.96 ± 0.14 respectively for each Ax-CCM group. Both the N13_E and N13_F were statistically different among Ax-CCM groups. (ANOVA, *p* < 0.001) A post hoc test showed that the N13_E for the type 1 pattern was significantly lower than that for the type 2 pattern (*p* < 0.05) (Fig. 4a).

19 patients were identified as cervical segmental instability in dynamic X-rays, and the rest 19 patients were identified as cervical stable. The N13_F varied significantly between patients in Stable and Unstable groups. (T-test, *p* = 0.007), but not for N13_E (T-test, *p* = 0.2) (Fig. 4b).

### The radiographic findings’ correlation with clinical data

**Fig. 5:**
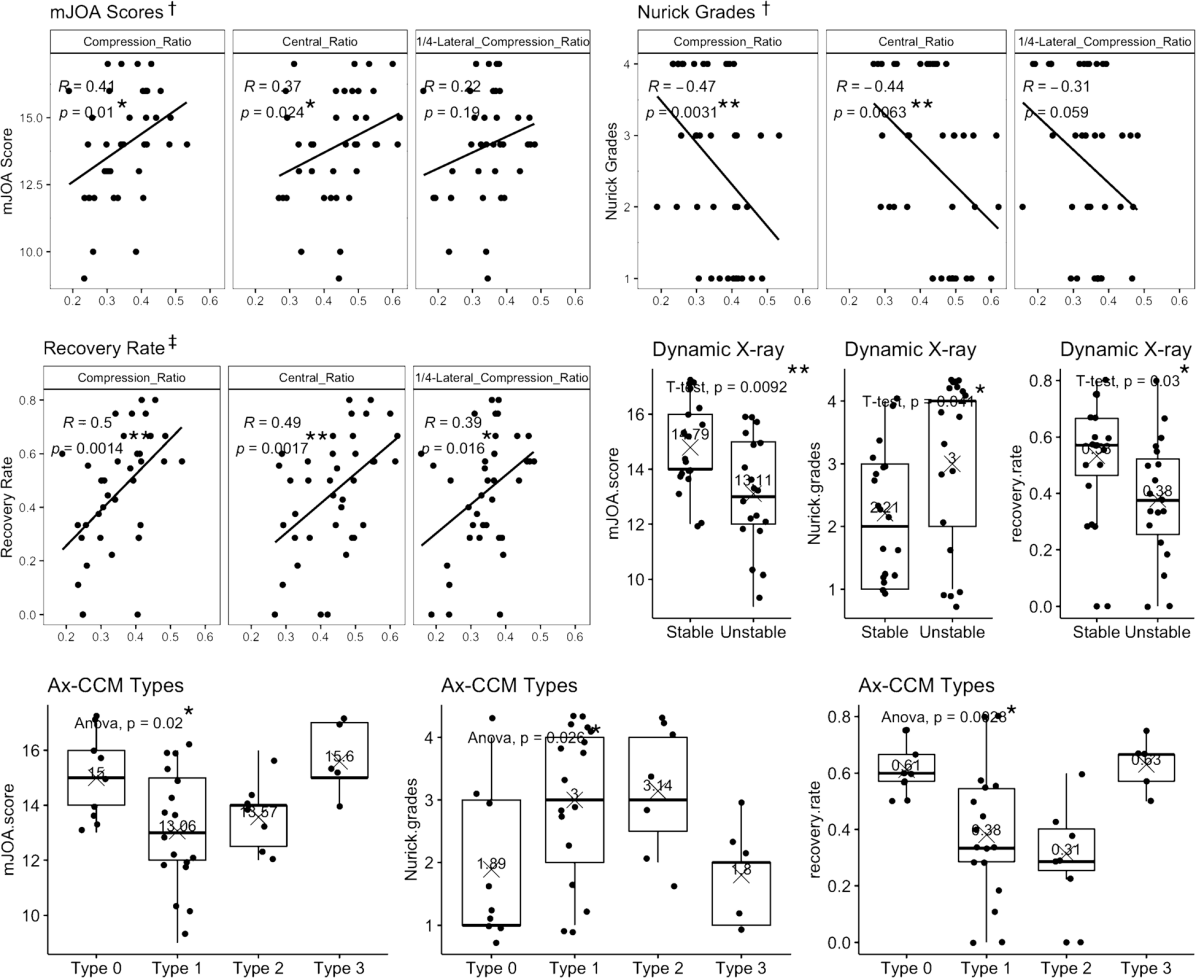
Correlations between radiographic measures and clinical data. * *p*_value< 0.05; ** *p*_value< 0.01; *** *p*_value< 0.001. ^†^ The mJOA and Nurick grades’ correlation with Compression_Ratio, Central_Ratio and 1/4-Lateral_Compression_Ratio were calculated with the Spearman method. ^‡^ The recovery rate’s correlation with Compression_Ratio, Central_Ratio and 1/4-Lateral_Compression_Ratio were calculated with the Pearson method

Both the Compression_Ratio and Central_Ratio measured in MRI were significantly correlated with the baseline mJOA scores (Spearman, *p* < 0.05), Nurick grades (Spearman, *p* < 0.01) and recovery rates (Pearson, *p* < 0.01). The 1/4-Lateral_Compression_Ratio was significantly correlated with the recovery rates (Pearson, *p* < 0.05), but not with baseline mJOA scores and Nurick grades. Patients’ mJOA scores, Nurick grades and recovery rates were all significantly different among different Ax-CCM groups (ANOVA, p < 0.05). The mJOA scores, Nurick grades and recovery rates in patients with or without cervical segmental instability were also significantly different (t-test, *p* < 0.05) (Fig. 5). All radiographic findings above did not vary between ataxic patients and patients without ataxia.

The original DSSEP, MRI measurement and clinical data of all 38 patients in the study can be found in the supplementary file.

## Discussions

The study first uncovered the dynamic neurophysiological changes behind MRI and dynamic X-ray findings in CSM patients. Patients with lower Compression_Ratio, Central_Ratio and 1/4-Lateral_Compression_Ratio in MRI and cervical segmental instability in dynamic X-ray showed more severe neurological dysfunction at extensions and flexions. Patients with diffuse intramedullary T2 hyperintensity with obscure and faint borders in MRI (Ax-CCM type 1) had their electro-neurophysiology most severely impacted at extension. While patients with focal intramedullary T2 hyperintensity and faint borders (Ax-CCM type 2) had their DSSEP changed most greatly at flexion. Our findings suggested that the compressive degree evaluated by the three mentioned MRI measurements and segmental instability evaluated by dynamic X-rays were associated with the extent of the dynamic neural deficit of the chronic compressive spinal cord. Patients’ different MRI intramedullary T2 hyperintensity patterns may suggest different modes of transient dynamic spinal cord injuries and distinct pathophysiology. These radiographic findings might have good potentials in aiding the diagnosis and prognostication of CSM clinically.

Our previous study has already proven the diagnostic effect of DSSEP N13 amplitude ratios [15]. Transient or reversible cervical cord dysfunction resulting from dynamic cervical cord compression may lead to the deterioration of some electrophysiological parameters, making DSSEP more sensitive and effective in diagnosing CSM than traditional static SSEP [15]. The DSSEP N13 potential mainly reflects signal transmittance through the cervical spinal cord to lower brainstem levels [21]. However, the Fz reference of the C2s-Fz montage may inject frontally recorded P14 wave into the N13 wave, rendering the N13 potential insensitive to central gray matter damage or dorsal column dysfunction [22, 23], which might be a flaw in this study. A better solution would be replacing the Fz reference with anterior cervical (AC) reference, in other words, replacing the C2s-Fz montage with C2s-AC montage [23]. Nevertheless, the N13 potentials recorded with C2s-Fz could still reflect abnormalities of the forehead P13 in neuro-transduction impaired CSM patients, and they were proved to correlate well with the preoperative mJOA score and Nurick grade in our study. The N20 recorded with a C4’-C3’ montage on the scalp, however, is a near-field potential sensitive to even a short-distance relocation of the electrode, and can be easily interfered by brain waves [21, 24]. This explains why the N13 amplitude is a more suitable parameter over the N20 amplitude in CSM evaluation. In this study, we used the DSSEP N13 amplitude ratios (N13_E and N13_F) to reflect transient cord dysfunction during motion in CSM patients. A lower value corresponded to a greater neurological change and a value of 0 indicated that the SSEP wave was absent. In other words, a smaller value may indicate either a serious, repetitive, or active micro-injury to the spinal cord during motion, or permanent damage to the cervical cord. On the contrary, a high value corresponded to less change in neurological function during movement, and suggested either a good neurological condition or a chronic and stable condition of the spinal cord. Both the N13_E and N13_F showed good correlations with our baseline clinical assessments and postoperative outcomes. Moreover, we observed a significant decrease in only the N13_E, rather than the N13_F in ataxic patients compared with patients without ataxia. The CSM-caused ataxia is associated with proprioceptive sensory deficit and dorsal columns involvement [18, 25], and is a predictor for poorer post-surgical outcome [26]. We assume extension movement can cause severer DSSEP decrease in ataxic patients, for it exerts more impingement on the posterior aspect of spinal cord compared with the flexion movement.

Compression of the spinal cord does not always cause clinical symptoms and it is difficult to infer the degree of dysfunction of the spinal cord from MRI findings. In this study, we used N13 DSSEP amplitude ratios as a tool for assessing the dynamic neurological deficit of CSM, and compared them with MRI measurements. We found both the N13_E and N13_F positively correlated with the Compression_Ratio, Central_Ratio and 1/4-Lateral_Compression_Ratio measured in MR axial images. Our results suggested that the extent of dynamic neural dysfunction of the cord was associated with the degree of spinal cord deformity caused by central canal stenosis as well as lateral compression. The Compression_Ratio and Central_Ratio are sensitive markers for myelopathy [27], which have been used regularly in the management of CSM. The 1/4-Lateral_Compression_Ratio has been proved to reflect the extent of dysfunction of the corticospinal tracts [28]. However, there was no correlation between the DSSEP results and the spinal cord sagittal diameter, area, or Cord/Canal Area Ratio. An explanation for it was that the spinal cord’s absolute sagittal diameter and cross-sectional area depend on several factors including height, cervical segmental level (greatest at C4), and age (greatest at the 3rd decade of life) [29–31], which vary substantially between individuals. The Cord/Canal_Area_Ratio failed to reflect the dynamic neural impact because it could not reflect the compression degree in some cases. For some patients with severe compression, the spinal cord and subarachnoid space may be flattened, with the spinal cord area and Cord/Canal Area Ratio remaining unchanged.

Intramedullary T2WI hyperintensity has been proved to be a sensitive marker for severe myelopathy and poor prognosis by many studies [32–34], and is thought to reflect a broad spectrum of pathologic changes from reversible edema and inflammation to irreversible vascular ischemia and cystic necrosis [35, 36]. However, not all T2WI signal changes are the same, and the changes appear in 2 broad forms depending on the degree of intensity deviation and the patterns of the signal change [37, 38]. The Ax-CCM grading system is based on the morphology and area of intramedullary hyper-intensity on axial T2WI and has been proved to correlate well with clinical pictures [8]. In this study, we used the Ax-CCM grading system to evaluate the CSM patients, and found both the N13_E and N13_F varied statistically (ANOVA, *p* < 0.05) among different Ax-CCM groups. The N13_E and N13_F in the Ax-CCM Type 1 and 2 groups were lower than that in the other two groups, suggested spinal cords in these groups were more vulnerable during motion. Interestingly, we found patients in Ax-CCM Type 1 group were most severely affected at extension, while the patients in Ax-CCM Type 2 group were at flexion. According to You et al., the type 1 pattern seems to indicate an acute, transient, and recuperative cord injury with relatively good circulation [8], making the spinal cord more vulnerable at extension because of the “pincer effect”, but relatively less affected at extension. The Ax-CCM type 2 pattern, or the so called “snake-eye appearance” in MRI, however, may indicate the occlusion of anterior radiculomedullary arteries and poorer spinal cord circulation in a previous CT angiography study [39]. The vasculature of the spinal cord is organized as such that its ventral aspect and grey matter is supplied centrifugally by the central artery and the anterior part of the vasocorona, both of which arise from the anterior spinal artery. As a result, patients in the Ax-CCM type 2 group tended to suffer greater neurological deterioration in the flexion position when their spinal cords are compressed from the ventral aspect where their anterior spinal arteries are compressed. Restuccia et al. reported that neck flexion causes a significant amplitude decrease of the N13 cervical response in patients with Hirayama disease [40]. In the disease, forward displacement of the cervical dural sac and compressive flattening of the lower cervical cord occurs during neck flexion [41]. This causes occlusion of the anterior spinal artery in the lower cervical spinal cord and subsequent ischemia in the anterior horn cells [41, 42]. If left untreated, Hirayama disease will result in lower cervical spinal cord atrophy and “snake-eye appearance” in MRI, which are considered negative prognostic indicators [42]. The exact pathophysiology of the cervical cords with different patterns of intramedullary T2 hyperintensity during motion still needs to be further studied.

We also found the N13_F were significantly lower among patients with cervical segmental instability, while the N13_E did not show a significant difference. We assume it was because patients with cervical segmental instability suffered greater subluxation and (or) rotation instability during flexion movements. During extension, the relative movements between the adjacent vertebrae of the unstable segment are generally milder, because the superior and inferior articular processes from adjacent vertebrae can help stabilize the cervical segment. Therefore, for CSM patients with segmental instability, cervical canal stenosis and neurological deteriorations mainly occurred at the flexion position.

Finally, we compared the radiographic data with baseline mJOA scores, Nurick grades and 2-year postoperative recovery rates. Compression_Ratio, Central_Ratio, 1/4-Lateral_Compression_Ratio, Ax-CCM types and cervical segmental instability in radiographs were statistically related to recovery rates. Apart from the 1/4-Lateral_Compression_Ratio, all these parameters also correlated with preoperative clinical assessments. These findings further testified the clinical utility of these radiographic parameters.

Our study had several limitations. Firstly, the reference electrode for the N13 should be located beyond the scalp, such as the anterior neck. We would modify our DSSEP method in our future studies. Secondly, this is a single-center retrospective study, which is limited in depth. A prospectively designed study with dynamic SSEPs is warranted to address this problem. Thirdly, not all patients received uniformed treatment, therefore we could not rule out other factors that different surgical methods may have on patient outcomes. Lastly, the sample size of the study was relatively small, limiting statistical power. In the future, we plan to expand the sample size to confirm the efficacy of our method.

## Conclusions

The spinal cord compression degree parameters including the Compression_Ratio, Central_Ratio and 1/4-Lateral_Compression_Ratio and the Ax-CCM classification system in MRI axial images as well as cervical segmental instability in dynamic X-ray corresponded to the dynamic neurological deficit of CSM. They could serve as substitutes to evaluate neurological damage in the absence of DSSEP to some extent, and reflect symptom severity and predict prognosis. Different intramedullary T2 hyperintensity patterns were associated with different DSSEP characteristics at extension and flexion, suggesting their differing pathophysiology.

## Supplementary information

**Additional file 1.**

## Data Availability

All data generated or analyzed during this study are included in this published article and its supplementary information files.

## Abbreviations

DSSEP: Dynamic somatosensory evoked potentials
SSEP: Somatosensory evoked potentials
CSM: cervical spondylotic myelopathy
N13_E: DSSEP N13 amplitude ratios at extension
N13_F: DSSEP N13 amplitude ratios at flexion
N20_E: DSSEP N20 amplitude ratios at extension
N20_F: DSSEP N20 amplitude ratios at flexion
MRI: Magnetic Resonance Imaging
Compression_Ratio: Compressive ratios at the compressed site in MRI axial images
Central_Ratio: Compressive ratios at central point in MRI axial images
1/4-Lateral_Compression_Ratio: Compressive ratios at 1/4-lateral points in MRI axial images
ANOVA: Analysis of variance
AC: Anterior cervical

## References

[1] Fukui K, Kataoka O, Sho T, Sumi M (1990). Pathomechanism, pathogenesis, and results of treatment in cervical spondylotic myelopathy caused by dynamic canal stenosis. Spine (Phila Pa 1976).

[2] Dai L, Ni B, Yuan W, Jia L (1998). Radiculopathy after laminectomy for cervical compression myelopathy. J Bone Joint Surg Br.

[3] Scheer JK, Tang JA, Smith JS, Acosta FL, Protopsaltis TS, Blondel B, Bess S, Shaffrey CI, Deviren V, Lafage V (2013). Cervical spine alignment, sagittal deformity, and clinical implications: a review. J Neurosurg Spine.

[4] Ames CP, Blondel B, Scheer JK, Schwab FJ, Le Huec JC, Massicotte EM, Patel AA, Traynelis VC, Kim HJ, Shaffrey CI (2013). Cervical radiographical alignment: comprehensive assessment techniques and potential importance in cervical myelopathy. Spine.

[5] Toledano M, Bartleson JD (2013). Cervical spondylotic myelopathy. Neurol Clin.

[6] Penning L, van der Zwaag P (1966). Biomechanical aspects of spondylotic myelopathy. Acta Radiol Diagn (Stockh).

[7] Nouri A, Martin AR, Mikulis D, Fehlings MG (2016). Magnetic resonance imaging assessment of degenerative cervical myelopathy: a review of structural changes and measurement techniques. Neurosurg Focus.

[8] You JY, Lee JW, Lee E, Lee GY, Yeom JS, Kang HS (2015). MR classification system based on axial images for cervical compressive myelopathy. Radiology.

[9] White AA, Johnson RM, Panjabi MM, Southwick WO (1975). Biomechanical analysis of clinical stability in the cervical spine. Clin Orthop Relat Res.

[10] Lu K, Gao X, Tong T, Miao D, Ding W, Shen Y (2017). Clinical predictors of surgical outcomes and imaging features in single segmental cervical Spondylotic myelopathy with lower cervical instability. Med Sci Monit.

[11] de Arruda Serra Gaspar MI, Cliquet A, Fernandes Lima VM, de Abreu DC (2009). Relationship between median nerve somatosensory evoked potentials and spinal cord injury levels in patients with quadriplegia. Spinal Cord.

[12] Morishita Y, Hida S, Naito M, Matsushima U (2005). Evaluation of cervical spondylotic myelopathy using somatosensory-evoked potentials. Int Orthop.

[13] Restuccia D, Di Lazzaro V, Lo Monaco M, Evoli A, Valeriani M, Tonali P (1992). Somatosensory evoked potentials in the diagnosis of cervical spondylotic myelopathy. Electromyogr Clin Neurophysiol.

[14] Nakai S, Sonoo M, Shimizu T (2008). Somatosensory evoked potentials (SEPs) for the evaluation of cervical spondylotic myelopathy: utility of the onset-latency parameters. Clin Neurophysiol.

[15] Qi Q, Huang S, Ling Z, Chen Y, Hu H, Zhan P, Zhang B, Zou X, Peng X. A new diagnostic medium for cervical spondylotic myelopathy: dynamic somatosensory evoked potentials. World Neurosurg. 2020;133:e225-e232. 10.1016/j.wneu.2019.08.205 31493599

[16] Benzel EC, Lancon J, Kesterson L, Hadden T (1991). Cervical laminectomy and dentate ligament section for cervical spondylotic myelopathy. J Spinal Disord.

[17] Nurick S (1972). The natural history and the results of surgical treatment of the spinal cord disorder associated with cervical spondylosis. Brain.

[18] Hwang WJ (2016). Reversible pseudoathetosis and sensory ataxic gait caused by cervical spondylotic myelopathy. J Clin Neurosci.

[19] Hirabayashi K, Miyakawa J, Satomi K, Maruyama T, Wakano K (1981). Operative results and postoperative progression of ossification among patients with ossification of cervical posterior longitudinal ligament. Spine (Phila Pa 1976).

[20] Sonoo M, Kobayashi M, Genba-Shimizu K, Mannen T, Shimizu T (1996). Detailed analysis of the latencies of median nerve somatosensory evoked potential components, 1: selection of the best standard parameters and the establishment of normal values. Electroencephalogr Clin Neurophysiol.

[21] Restuccia D, Di Lazzaro V, Valeriani M, Tonali P, Mauguiere F (1992). Segmental dysfunction of the cervical cord revealed by abnormalities of the spinal N13 potential in cervical spondylotic myelopathy. Neurology.

[22] Restuccia D, Mauguiere F (1991). The contribution of median nerve SEPs in the functional assessment of the cervical spinal cord in syringomyelia. A study of 24 patients. Brain.

[23] Mauguiere F, Restuccia D (1991). Inadequacy of the forehead reference montage for detecting abnormalities of the spinal N13 SEP in cervical cord lesions. Electroencephalogr Clin Neurophysiol.

[24] Nuwer MR, Packwood JW. Somatosensory evoked potential monitoring with scalp and cervical recording. In: Daube JR, Francois M, editors. Handbook of Clinical Neurophysiology. Volume 8. Amsterdam: Elsevier; 2008. p. 180–9.

[25] Koeppen AH (2011). Friedreich's ataxia: pathology, pathogenesis, and molecular genetics. J Neurol Sci.

[26] Tetreault LA, Kopjar B, Vaccaro A, Yoon ST, Arnold PM, Massicotte EM, Fehlings MG (2013). A clinical prediction model to determine outcomes in patients with cervical spondylotic myelopathy undergoing surgical treatment: data from the prospective, multi-center AOSpine North America study. J Bone Joint Surg Am.

[27] Harrop JS, Naroji S, Maltenfort M, Anderson DG, Albert T, Ratliff JK, Ponnappan RK, Rihn JA, Smith HE, Hilibrand A (2010). Cervical myelopathy: a clinical and radiographic evaluation and correlation to cervical spondylotic myelopathy. Spine (Phila Pa 1976).

[28] Kanchiku T, Taguchi T, Kaneko K, Fuchigami Y, Yonemura H, Kawai S (2001). A correlation between magnetic resonance imaging and electrophysiological findings in cervical spondylotic myelopathy. Spine (Phila Pa 1976).

[29] Ishikawa M, Matsumoto M, Fujimura Y, Chiba K, Toyama Y (2003). Changes of cervical spinal cord and cervical spinal canal with age in asymptomatic subjects. Spinal Cord.

[30] Kato F, Yukawa Y, Suda K, Yamagata M, Ueta T (2012). Normal morphology, age-related changes and abnormal findings of the cervical spine. Part II: magnetic resonance imaging of over 1,200 asymptomatic subjects. Eur Spine J.

[31] Sherman JL, Nassaux PY, Citrin CM (1990). Measurements of the normal cervical spinal cord on MR imaging. AJNR Am J Neuroradiol.

[32] Mastronardi L, Elsawaf A, Roperto R, Bozzao A, Caroli M, Ferrante M, Ferrante L (2007). Prognostic relevance of the postoperative evolution of intramedullary spinal cord changes in signal intensity on magnetic resonance imaging after anterior decompression for cervical spondylotic myelopathy. J Neurosurg Spine.

[33] Nouri A, Tetreault L, Zamorano JJ, Dalzell K, Davis AM, Mikulis D, Yee A, Fehlings MG (2015). Role of magnetic resonance imaging in predicting surgical outcome in patients with cervical spondylotic myelopathy. Spine (Phila Pa 1976).

[34] Shin JJ, Jin BH, Kim KS, Cho YE, Cho WH (2010). Intramedullary high signal intensity and neurological status as prognostic factors in cervical spondylotic myelopathy. Acta Neurochir.

[35] Mizuno J, Nakagawa H, Inoue T, Hashizume Y (2003). Clinicopathological study of "snake-eye appearance" in compressive myelopathy of the cervical spinal cord. J Neurosurg.

[36] Mizuno J, Nakagawa H, Chang HS, Hashizume Y (2005). Postmortem study of the spinal cord showing snake-eyes appearance due to damage by ossification of the posterior longitudinal ligament and kyphotic deformity. Spinal Cord.

[37] Tetreault LA, Dettori JR, Wilson JR, Singh A, Nouri A, Fehlings MG, Brodt ED, Jacobs WB (2013). Systematic review of magnetic resonance imaging characteristics that affect treatment decision making and predict clinical outcome in patients with cervical spondylotic myelopathy. Spine (Phila Pa 1976).

[38] Vedantam A, Rajshekhar V (2013). Does the type of T2-weighted hyperintensity influence surgical outcome in patients with cervical spondylotic myelopathy? A review. Eur Spine J.

[39] Zhang Z, Wang H (2014). Is the "snake-eye" MRI sign correlated to anterior spinal artery occlusion on CT angiography in cervical spondylotic myelopathy and amyotrophy?. Eur Spine J.

[40] Restuccia D, Rubino M, Valeriani M, Mirabella M, Sabatelli M, Tonali P (2003). Cervical cord dysfunction during neck flexion in Hirayama's disease. Neurology.

[41] Hirayama K, Tokumaru Y (2000). Cervical dural sac and spinal cord in juvenile muscular atrophy of distal upper extremity. Neurology.

[42] Xu H, Shao M, Zhang F, Nie C, Wang H, Zhu W, Xia X, Ma X, Lu F, Jiang J (2019). Snake-eyes appearance on MRI occurs during the late stage of Hirayama disease and indicates poor prognosis. Biomed Res Int.

